# Modulating the 3’ end-DNA and the fermentation process for enhanced production and biological activity of porcine interferon-gamma

**DOI:** 10.1371/journal.pone.0214319

**Published:** 2019-03-26

**Authors:** Haiming Cai, Jinbo Deng, Jiaoqing Li, Miaopeng Ma, Chaoyuan Huang, Peijing Zhao, Feiping Ming, Qianyi Liang, Junhao Jia, Shuxia Zhang, Min Zeng, Linghua Zhang

**Affiliations:** Guangdong Provincial Key Laboratory of Protein Function and Regulation in Agricultural Organisms, College of Life Sciences, South China Agricultural University, Guangzhou, Guangdong, China; Instituto Butantan, BRAZIL

## Abstract

Porcine gamma interferon is a cytokine produced by activated T cells and NK cells with broad-spectrum antiviral activity and immunomodulatory function. However, pIFN-γ is a secretory protein that has a short half-life in organisms and is easily inactivated, making it difficult to apply widely in clinics. Therefore, we tried to optimize the expression of pIFN-γ in *Pichia pastoris* to obtain a large amount of highly active, easily purified pIFN-γ protein in vitro. Through C-terminal sequence analysis, we found a signal sequence (EKREAEAE) that was easily enzymolysed by a signal peptide enzyme, resulting in degradation and inactivation of the pIFN-γ protein. In this study, we optimized the *pIFN-γ* gene recombination sequence and mutated the 3' end of the *pIFN-γ* gene, resulting in a higher expression level and stronger biological activity, as well as a significant upregulation in the expression of the interferon-stimulated genes *Mx1* and *OAS1* in IPEC-J2 jejunal epithelial cells. Our data also showed that the fermentation process could significantly improve productivity. A recombinant *Pichia pastoris* strain with the optimized *pIFN-γ* gene could obtain a high yield of pIFN-γ protein, up to 9536 mg/L, after staged incubation for 0–24 h at 28°C, pH 6.0, and 50% dissolved oxygen (DO), followed by incubation for 24–72 h at 25°C, pH 6.0 and 30% DO. These data demonstrated, for the first time, that the expression level of pIFN-γ in *Pichia pastoris* was improved significantly by gene optimization with 3' end mutation and a fermentation process that maintained good biological activity, which is beneficial to the application of pIFN-γ in animal husbandry.

## Introduction

Interferon gamma (IFN-γ) is a cytokine with antiviral and immunomodulatory functions produced by activated T cells and NK cells[1–3]. The total porcine interferon gamma IFN-γ (pIFN-γ) gene is 501 bases and encodes 166 amino acids. Twenty-three amino acids compose the signal peptide, and 143 amino acids of pIFN-γ function in the activity of the mature protein[4]. pIFN-γ mainly participates in the immune regulation effect by activating the body's own immunity against viral attack[5]. The antiviral effect of interferon does not directly kill the virus but rather binds to cell surface receptors[6] to induce cells to produce enzyme-active antiviral proteins ADAR, PKR, Mx, OAS, and RNaseL, which degrade viral RNA, inhibit viral replication and translation, and inhibit viral shell formation to achieve antiviral effects[7–9]. Studies have shown that pIFN-γ can significantly inhibit the proliferation of reproductive and respiratory syndrome virus (PRRSV)[6] and plays an important role in anti-swine fever virus (CSFV) cellular immunity[10]. A study also showed that pIFN-γ could enhance the immune response of piglets to dysentery antigens and played an important role in anti-Salmonella infection. pIFN-γ has broad application prospects due to its highly effective antiviral and immunomodulatory effects. The researchers also used different expression systems to express pIFN-γ, but most of them have a problem with low protein expression or activity, which limits the further application of pIFN-γ[11].

Under normal circumstances, the interferon species in animals is highly specific, has low expression levels and is difficult to extract and purify, Wang Li et., al studies found that *E*.*coli*, *Baculovirus* can improve the co-expression level of interferon, while the expression products need to undergo degeneration, complex re-purification, which were seriously affecting the biological activity of interferon [11–13], so research and development of a biological system for efficient production of interferon has aroused the attention of scholars globally. pIFN-γ was first expressed in *Escherichia coli* (*E*. *coli*) by genetic engineering, and with the improvement of extraction process, the pIFN-γ protein extraction rate reached 95% ~ 97%, while the other 3% ~ 5% was *E*. *coli* protein, which could easily cause substantial side effects such as fever, allergy and other adverse reactions. To express recombinant pIFN-γ with high efficiency, low cost and few side effects, in recent years, research on the expression system of methanolic yeast has progressed rapidly[14]. This system is regulated by the methanol-regulated alcohol oxidase 1 (*AOX1*) promoter, which strictly controls the expression of exogenous genes[15] and has high stability and high secretion. The host bacterium *Pichia pastoris* has little endogenous protein secretion but highly active exogenous protein secretion, which is beneficial for downstream separation and purification operations and large-scale fermentation production; therefore, *Pichia pastoris* is a suitable eukaryotic expression system for exogenous genes[16]. In our previous studies, we found that interferon could not be purified by nickel-column affinity chromatography. By analysis of the 3’ end of the gene, we found that the amino acids in positions 126–132 of the C-terminus may be similar to the cleavage site of the secreted α signal peptide before its secretion. C-terminal peptides would be cleaved, and pIFN-γ protein with a complete C-terminus would fail to be secreted. After the optimization of the 3’-peptide chain, a large increase in biological activity was observed in yeast. In addition, it is well known that the fermentation process, especially the fermentation tank technology, is very important for the production of foreign proteins secreted by *Pichia pastoris*. *Pichia pastoris* does not affect normal growth and metabolism due to the accumulation of fermentation products. It is also easy to expand from shake flask culture to high density fermentation without affecting the expression level of exogenous genes, so it has great potential for high density fermentation; however, the expression system of *Pichia pastoris* has a great difference in the expression of different exogenous proteins, reaching up to 12 g/L or as low as only 1 mg/L, a 10,000-fold difference[17]. This difference in expression of the foreign protein stems, on the one hand, from the characteristics of the foreign gene itself, and on the other hand, from the fermentation conditions, with the latter having an important influence. The effect of temperature on the activity of yeast fermentation products, the physical properties of fermentation broth and the direction of biosynthesis, as well as the DO in the fermentation matrix, are very important to the growth of aerobic microorganisms. Therefore, in this study, we used the *Pichia pastoris* eukaryotic expression system, regulating the 3’ end-DNA and fermentation process for enhanced production and biological activity of pIFN-γ in recombinant *Pichia pastoris*, to ensure the high-level expression of the protein at the transcription and translation levels, which laid a foundation for the construction of high-yield strains of pIFN-γ *Pichia pastoris*. The use of secretory expression greatly simplifies the subsequent purification steps and costs of the target protein and facilitates its industrial production.

## Materials and methods

### Strains and plasmids

Strains *P*. *pastoris* X33, *E*. *coli* DH5α, and pPICZαA were purchased from Invitrogen (CA, USA). The IPEC-J2 cell line (small intestinal epithelial cell) was a gift from Deng Yiqun, South China Agricultural University.

### Synthesis of the *pIFN-γ* gene and construction of the expression vector

To improve the production of recombinant pIFN-γ in *P*. *pastoris*, we used a novel deterministic computational algorithm COStar for codon optimization[18]. The optimal synthetic sequence, *pIFN-γ*_*1*_ (GenBank: MH513659), had the following properties: enhanced codon usage bias of the heterologous gene; no unwanted cleavage sites for restriction enzymes and negative cis-acting elements; a reduced number of highly repetitive nucleotide sequences; adaptive G+C content; and inhibited local formation of the transcribed mRNA secondary structure. We designed a cloning approach based on synthetic overlapping primers and a PCR assembly strategy to construct a synthetic pIFN-γ fragment without the signal peptide sequence (GenBank: NM_213948). The pIFN-γ cDNA (GenBank: NP_999113) was used as a template for optimization to generate a set of twelve overlapping oligonucleotides. A mixture of the oligonucleotides was used in the first-round PCR procedure consisting of ten cycles, using KOD-FX polymerase to generate the coding sequence. Then, the region for the *Xho*Ⅰ restriction site and *KEX*2 cleavage site of the α-factor signal sequence of *P*. *pastoris* were added to the 5’ terminus of the synthesized fragment in the second-round PCR using the primer pIFN-γ-forward. At the same time, the region for the *Xba*Ⅰ restriction site was linked to the 3’ terminus using the primer pIFN-γ-reverse, which was inserted into the expression vector pPICZαA to construct the plasmid pPICZαA-pIFN-γ_1_ (S1 Fig). The other region for the *Xba*Ⅰ restriction site was linked to the 3’ terminus using the primer pIFN-γ-reverse with six histidine (His) tags. The complete artificial DNA was inserted into the expression vector pPICZαA to construct the plasmid pPICZαA-pIFN-γ_1_-His (S2 Fig).

The arginine (R) at positions 129 and 131 of the pIFN-γ mature peptide chain was mutated to a histidine (H); that is, the amino acid sequence of the peptide chain from 126 to 132 was mutated from LRKRKRS to RKHKH, and accordingly, the corresponding codon was mutated from AGA and CGT to CAT (S3 Fig). Then, we used a novel deterministic computational algorithm, COStar, for codon optimization of the *pIFN-γ*_1_’ gene (GenBank: MH513659)[18]. Through comprehensive analysis and optimization, the full-length pIFN-γ gene with signal peptide mutation was obtained. The obtained fragment was inserted into the pPICZαA vector to construct two mutant secretory expression vectors pPICZαA-pIFN-γ_1_’ and pPICZαA-pIFN-γ_1_’-His tag.

*E*. *coli* DH5α was used for the construction of the recombinant expression plasmid and standard plasmid. All plasmids were confirmed by DNA sequencing, and the plasmids used in this study are shown in S1 Table and S4 Fig. All primers are listed in S2 Table.

### Transformation of *P*. *pastoris* and screening of transformants

*P*. *pastoris* X33 electrocompetent cells were transformed with *SacI*-linearized pPICZαA-pIFN-γ_1_, pPICZαA-pIFN-γ_1_’, pPICZαA-pIFN-γ_1_-His tag, or pPICZαA-pIFN-γ_1_’-His tag. Positive transformants were screened on YPDS containing 500 μg/ml zeocin. Transformants confirmed by PCR were grown overnight in BMGY in preparation for use as competent cells, and they were used for a second round of electroporation. The transformation mix was spread on BMGY plates containing 500 or 1000 μg/ml zeocin for direct selection of potential multicopy recombinants.

### Small-scale pIFN-γ expression and purification

Colonies of transformants were cultivated in BMGY culture medium containing 100 mM potassium phosphate (pH 6.0), 1.34% yeast nitrogen base without amino acids, 4 × 10^−5^% biotin, and 1% glycerol (BMGY). Cultures were grown in 500 ml flasks containing 50 ml of BMGY and were incubated at 28°C on a rotary shaker at 230 rpm. When the OD_600_ reached 5–6, the cells were centrifuged at 3500 rpm for 5 min, and the cell pellet was resuspended in 50 ml of BMMY. To maintain the expression of the product, 100% methanol was added to a final concentration of 0.5% (v/v) every 24 h. After 48 h of induction, 20 μl of culture supernatant was analyzed by SDS-PAGE. To purify the pIFN-γ protein in the supernatant, the culture supernatant was dialyzed against 50 mM Tris-HCl, pH 7.5. The cation exchanger CM-Sephadex was pre-equilibrated with 50 mM Tris-HCl, pH 7.5, and then the culture supernatant was packed in a column and washed with the same buffer until the absorbance reached <0.1 at 280 nm. After this step, pIFN-γ protein was eluted with a stepwise gradient of NaCl (0.1, 0.4, 0.6, 0.8, and 1 M NaCl prepared in 50 mM Tris-HCl, pH 7.5). The eluted fractions containing pIFN-γ were pooled and dialyzed against 5 mM Tris-HCl (pH 7.5) with 150 mM NaCl, 5 μM ZnSO_4_, and 5% glycerol[19] for further studies.

### Extraction of intracellular proteins from *P*. *pastoris*

Protein extractions from cytoplasmic and membrane-associated fractions were performed according to Shen et al.[20]. Briefly, cells were harvested with 1.5 × 10^8^ cells washed in PBS pH 7.4 and resuspended in 300 μl of yeast breaking buffer. An equal volume of acid-washed glass beads was added, and cells were disrupted by vortexing ten times for 1 min with 1-min intervals on ice. The lysate was centrifuged at 10,000×g for 30 min at 4°C, and the supernatant was collected. The pellet was further resuspended in 100 μl of yeast breaking buffer plus 2% SDS. After centrifugation at 4000×g for 5 min at 4°C, the supernatants containing the membrane-associated proteins were collected. Twenty micrograms of cytoplasmic proteins or membrane-associated proteins determined by BCA protein assay was analyzed by SDS-PAGE and Western blot.

### SDS-PAGE analysis and Western blot assays

Equal amounts of extracted proteins and 20 μl of culture medium were analyzed by 15% SDS polyacrylamide gel electrophoresis (PAGE); after electrophoresis, gels were stained with Coomassie blue R-250. For the Western blot assay, the proteins were transferred to PVDF membrane, and the membrane was incubated at 37°C with 5% nonfat milk. Afterward, the membrane was incubated with a 1:2000 dilution of goat anti-PGLYRP-1 polyclonal antibody (Santa Cruz, USA) overnight at 4°C and incubated with HRP conjugated rabbit polyclonal anti-goat IgG (CWBIO, China) at a dilution of 1:4000. Immunoreactive bands were visualized with the Enhanced HRP-DAB Chromogenic Substrate Kit (TIANGEN, China).

### Optimization of fermentation conditions of pIFN-γ in *Pichia pastoris*

#### Shake flask fermentation at different temperatures

A positive clone of a single colony was scraped from the BMGY plate and placed in 300 ml shake flasks containing 50 ml of seed medium (YPD) at 28°C, pH 6.0, and 200 rpm. The strains were cultured for 16–24 h, and then the seeds were transferred to shake flasks and fermented at 15°C, 20°C, 25°C and 30°C at pH 6.0 for the next 48 h[21].

#### Five liter fermenter with different DO levels

The liquid culture volume was 60% (vv-1), the inoculation volume was 10% (vv-1)[22], and the stirring speed was 150 rpm, with 50% DO at 28°C for 24 h. Then, the fermentation temperature was reduced to 25°C and the culture was continued from 24 h to 72 h (based on data of shake flask fermentation at different temperatures), with the DO at 20%, 30%, 40% and 50%[23]. For determination of the recombinant pIFN-γ protein concentration, the supernatant was obtained by centrifugation of the fermentation broth at 10,000 rpm for 10 min and subjected to SDS-PAGE gel electrophoresis.

The electrophoretic gel was scanned by gel imaging and analyzed by BandScan 5.0 software to calculate the protein concentration.

The wet weight of yeast was determined by removing 10 ml of fermentation liquid, centrifuging twice at 1000 rpm for 5 min, and weighing the yeast.

### Analysis of biological activity of pIFN-γ

The IPEC-J2 cell line, a gift from Deng Yiqun, South China Agricultural University, was maintained in Dulbecco’s MEM nutrient mix F12 (DMEM-F12) (1:1) (Gibco, Guangzhou, China) with 10% (v/v) FCS (Gibco, Guangzhou, China) at 37°C in 5% CO_2_. Prior to the treatment, IPEC-J2 cells were resuspended by trypsinization with 0.25% trypsin-EDTA and seeded into 24-well plates at 2×10^5^ cells/well without antibiotics. The concentration of the cell suspension was 5×10^4^/ml, and 100 μL of cell culture medium was added to each well. The cells were inoculated into 6 wells and cultured for 24 h at 5% CO_2_ at 37°C. After cell adherence, pIFN-γ_1_, pIFN-γ_1_-His, pIFN-γ_1_’, and pIFN-γ_1_’-His were added to a final concentration of 10 μg/3 mL. Then, the cells were collected, and total RNA was extracted from the cells.

### Quantitative real-time PCR assays

Total RNA was extracted from IPEC-J2 cells using the TRIzol method. RNA integrity was checked on 1% agarose gels and quantified using NanoDrop (Thermo Scientific, USA). After heating at 85°C for 30 min to denature RNA, 500 ng of total RNA was subjected to reverse transcription using the ReverTra Ace quantitative real-time PCR (qPCR) RT Kit (TOYOBO, Japan). Expression of mRNA was quantified with qPCR using a commercial reagent kit. For each of the targeted genes, a pair of oligonucleotide primers was designed using Primer Premier 5.0 software based on the sequences registered in the GenBank database (GenBank accession number: OAS1: XM_021073680.1, Mx1: NM_214061.2, PKR: KU212868, and actin: AF216956). Values for each target gene were normalized using actin. Expression values were calculated using the 2^-△△Ct^ method. The copy numbers of the *OAS1*, *Mx1*, and *PKR* genes in each strain were estimated according to the published method with modifications. The actin sequence was used as an endogenous gene, while *OAS1*, *Mx1*, and *PKR* sequences were used as target genes.

### Statistical analysis

Data were analyzed using the Statistical Analysis System (SAS 9.1.3). Differences in expression levels were investigated using one-way analysis of variance (ANOVA). Means of the values were compared using Duncan’s multiple comparison tests. A *p* value of <0.01 was considered significant.

## Results

### Construction of expression plasmids and transformation of *P*. *pastoris*

#### Construction of expression plasmids

**Fig 1:**
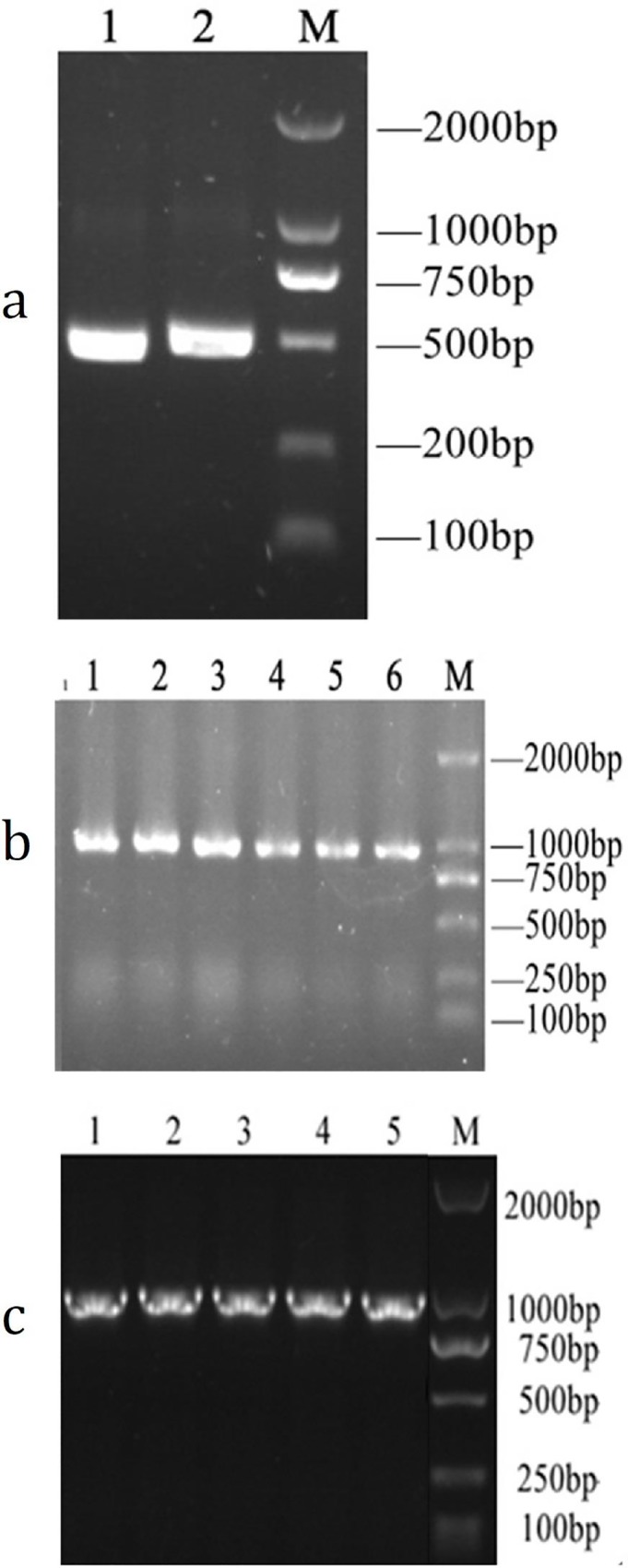
Construction of expression plasmids and transformation of *P*. *pastoris*. (a)Amplification of pIFN-γ gene with high fidelity PCR after optimization. M 3452 wide-type protein standard molecular weight marker, Lane 1–2 for optimized pIFN-γ_1_ gene. (b) Transformation of pIFN-γ_1_ and pIFN-γ_1_-His tag in *P*. *pastoris*. M 3452 wide-type protein standard molecular weight marker, Lane1-3 for pPICZαA- pIFN-γ_1_-His tag, Lane 4–6 for pPICZαA- pIFN-γ_1_. (c) Transformation of pPICZαA-pIFN-γ_1_’and pPICZαA-pIFN-γ_1_’-His tag in *Pichia pastoris*. M for DL2000 marker, Lane 1–2 for pPICZαA- pIFN-γ_1_’-His tag, Lane 3–5 for pPICZαA- pIFN-γ_1_’. (pIFN-γ_1_: a modified porcine gamma by yeastcodon preference; pIFN-γ1-His tag:a modified porcine gamma (pIFN-γ_1_)with adding histidine label; pIFN-γ_1_’:a modified pIFN-γ (pIFN-γ_1_)with C-terminal amino acid mutation; pIFN-γ_1_’-His tag:a modified pIFN-γ (pIFN-γ_1_)with C-terminal amino acid mutation and adding histidine label).

*pPICZαA-pIFN-γ*_*1*_ *and pPICZαA-pIFN-γ*_*1*_*-His tag in P*. *pastoris*. COStar software was used to optimize the encoding sequence of *pIFN-γ*, and the optimized *pIFN-γ*_*1*_ gene (GenBank: MH513659) was obtained by overlapping PCR (Fig 1A) with a molecular weight of 500 bp. The optimized results were that the base sequence similarity between the optimized sequence and the original sequence was 70.35%, and the G/C value was 43.0%. The full-length *pIFN-γ*_*1*_ gene and pIFN-γ_1_-His tag were obtained by overlapping PCR, and the plasmids were synthesized by Sangon Biotechnology. Nucleotide sequencing analysis confirmed that the plasmids contained the correct gene. Then, the recombinant strains containing *pIFN-γ*_*1*_ and *pIFN-γ*_*1*_-His were generated by transformation of *P*. *pastoris* X33 with the linearized vector pPICZαA (Fig 1B). The molecular weight of the carrier was approximately 1000 bp.

#### Construction of 3’ C-terminal signal peptide mutation expression plasmids pPICZαA-pIFN-γ_1_’ and pPICZαA-pIFN-γ_1_’-His tag in *P*. *pastoris*

The 129th and 131st arginine (R) of the previously optimized *pIFN-γ*_*1*_ gene signaling peptide were mutated to histidine (H), the peptide chain from positions 126 to 132 of the amino acid sequence was mutated from LRKRKRS to PKHKH, and the corresponding codon was mutated from AGA and CGT to CAT. Two mutant secretory expression vectors were constructed, named pPICZαA-pIFN-γ_1_**’** and pPICZαA-pIFN-γ_1_**’**-His tag. The plasmids were synthesized by Sangon Biotechnology. Nucleotide sequencing analysis confirmed that the plasmids contained the correct gene. Then, the recombinant strains containing *pIFN-γ*_*1*_***’*** and *pIFN-γ*_*1*_*’*-His tag were generated by transformation of *P*. *pastoris* X33 with the linearized vector pPICZαA (Fig 1C). The molecular weight of the carrier was approximately 1000 bp.

### Protein expression of pIFN-γ interferon in *Pichia pastoris*

**Fig 2:**
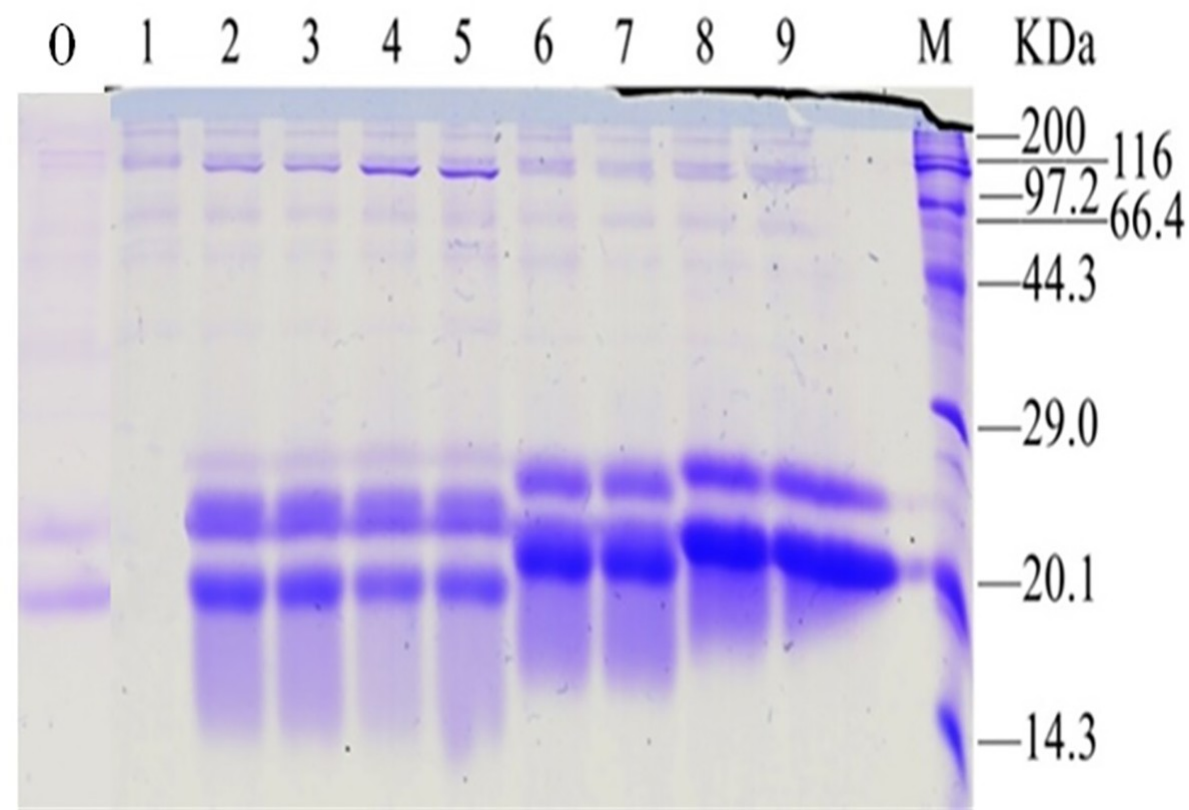
Protein expression of pIFN-γ in *P*. *pastoris*. M 3452 wide-type protein standard molecular weight marker, Lane 0 for pIFN-γ, Lane 1 for X33 strains, Lane 2–3 for pIFN-γ-His tag, Lane 4–5 for pIFN-γ protein in *P*. *pastoris*, Lane 6–7, pIFN-γ_1_’ protein in *P*. *pastoris*, Lane 8–9, pIFN-γ_1_’-His tag protein in *P*. *pastoris*. (pIFN-γ: for unoptimized pIFN-γ; pIFN-γ_1_: a modified porcine gamma by yeastcodon preference; pIFN-γ_1_-His tag: a modified pIFN-γby yeast codon preference(pIFN-γ_1_)with adding histidine label; pIFN-γ_1_’: a modified pIFN-γ (pIFN-γ_1_)with C-terminal amino acid mutation; pIFN-γ_1_’-His tag: a modified pIFN-γ (pIFN-γ_1_)with C-terminal amino acid mutation and adding histidine label).

The recombinant strains containing *pIFN-γ*_*1*_, *pIFN-γ*_*1*_-His tag, *pIFN-γ*_*1*_***’*** and *pIFN-γ*_*1*_***’****-*His tag in *P*. *pastoris* X33 were induced in BMGY (48 h, 28°C, 230 rpm), and the bacteria were collected. The expression of pIFN-γ protein was detected by SDS-PAGE. Data are shown in Fig 2. The recombinant strains of *Pichia pastoris* X33 containing *pIFN-γ*_*1*_, *pIFN-γ*_*1*_-His tag, *pIFN-γ*_*1*_***’*** and *pIFN-γ*_*1*_***’****-*His tag all expressed pIFN-γ. After *pIFN-γ* gene optimization, the expression level of protein in *Pichia pastoris* increased significantly compared with unoptimized *pIFN-γ* (*p*<0.05), especially after the 3’ C-terminal mutation optimized *pIFN-γ*_*1*_ (*p*<0.01). From the electrophoresis results (Fig 2), the molecular weight relationships of expressed pIFN-γ proteins were as follows: pIFN-γ_1_**’**-His tag> pIFN-γ_1_**’**> pIFN-γ_1_-His tag≈pIFN-γ_1_≈pIFN-γ (16.8 kDa).

### Western blot identification and nickel column purification of pIFN-γ protein with 6 histidine tags

**Fig 3:**
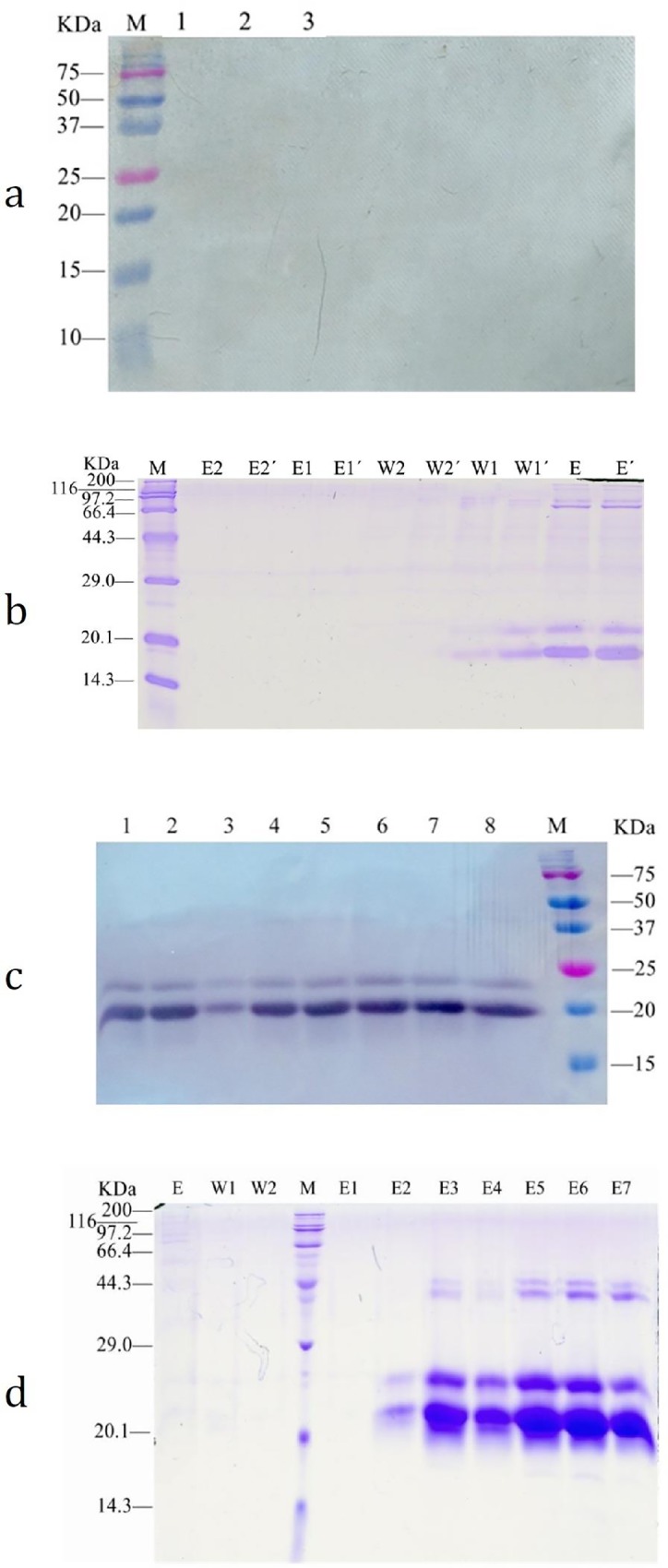
Western-blot identification and purification of pIFN-γ protein. (a)The western-blot of pIFN-γ_1_-His tag protein M for 3452 wide-type protein standard molecular weight marker, Lane 1–3 for induction of fermentation supernatant of x33-pIFN-γ-His tag strains. (b) Purification of pIFN-γ1-His tag protein by nickel column M a standard protein marker, E, E´ liquid for fermentation supernatant and nickel column after the combination of outflow fluid W1, W1´, W2, W2´ as 20 mM imidazole concentration of wash buffer elution, E1, E1´, E2, E2´ as 250 mM imidazole concentrations elution buffer wash off liquid. (c) Detection pIFN-γ_1_’-His tag by western-blot Lane 1–8 x33-pIFN-γ_1_’-His tag *P*.*pastoris* strain induced expression fermentation supernatant, M 3452 wide-type protein standard molecular weight marker. (d) Nickel column Purification analysis of the recombinant *P*. *pastoris* supernatant expressing pIFN-γ_1_’-His tag. M a standard protein marker, E, E´ liquid for fermentation supernatant and nickel column after the combination of outflow fluid W1, W1´, W2, W2´ 20 mM imidazole concentration of wash buffer elution, E1, E1´, E2, E2´ as 250 mM imidazole concentrations elution Buffer wash off liquid. (pIFN-γ_1_-His tag: a modified pIFN-γ by yeast codon preference (pIFN-γ_1_) with adding histidine label; pIFN-γ_1_’-His tag: a modified pIFN-γ (pIFN-γ_1_) with C-terminal amino acid mutation and adding histidine label).

The results showed that pIFN-γ_1_-His tag could secrete pIFN-γ protein in *Pichia pastoris*, while Western blot electrophoresis and nickel column purification could not detect the target protein (Fig 3A and 3B). However, Western blot analysis successfully detected the complete pIFN-γ peptide chain secreted by pIFN-γ_1_**’**-His tag in *Pichia pastoris* (Fig 3C), which had significant differences compared with the pIFN-γ_1_-His tag (*p*<0.01). Purification on a Ni-column after expression of the x33-pIFN-γ_1_**’**-His tag yeast strain fermented with 1 mg/ml zeocin (Fig 3D) was successful in obtaining the target protein pIFN-γ, with a content of up to 536.4 mg/ml.

From the above results, the expression of the *pIFN-γ* gene in *Pichia pastoris* was significantly increased after gene optimization and signaling peptide-amino acid mutation.

### Optimization of fermentation conditions of pIFN-γ in *Pichia pastoris*

#### Shake flask fermentation at different temperatures in *Pichia pastoris*

**Fig 4:**
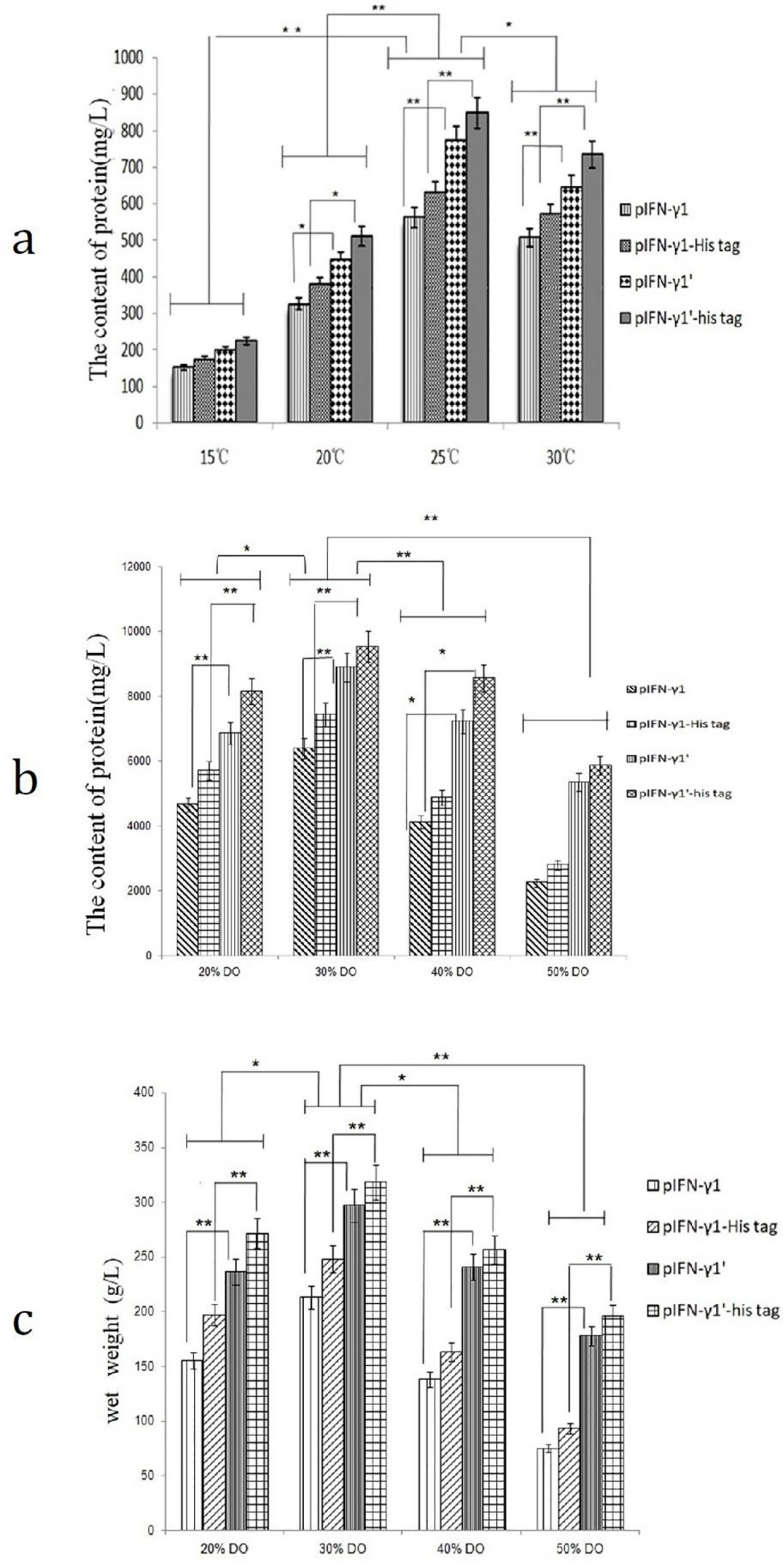
Optimization of secretion conditions of pIFN-γ protein in *P*. *pastoris*. (a) Effect of temperature on expression of pIFN-γ protein in *P*. *pastoris* by Shake flask fermentation. Recombinant pIFN-γ *Pichia pastoris*, at 28°C, pH 6.0, cultured 24 h, then 15°C, 20°C, 25°C, 30°C, pH 6 for 48 h, the results of the independent experiments were indicated as mean ±SD. * p<0.05 **p<0.01 indicated significant difference vs. corresponding group (see “Materials and methods”). (b) Effect of DO content of pIFN-γ protein in *P*. *pastoris* by fermentation of 5L fermentation tank. Recombinant pIFN-γ *Pichia pastoris*, at 28°C, pH 6.0, 50% DO cultured 24 h, then 25°C, pH 6, 30% DO, for the next 48 h, the results of the independent experiments were indicated as mean ±SD. * p<0.05 **p<0.01 indicated significant difference vs. corresponding group (as shown in“Materials and methods”). (c) Effect of DO content of pIFN-γ protein in *P*. *pastoris* by fermentation of 5L fermentation tank (at 28°C, pH 6.0, cultured 24 h, then 15°C, 20°C, 25°C, 30°C, pH 6 for 48 h). The figure showed the change of wet weight of yeast cells after different DO fermentation. The results of the independent experiments were indicated as mean ±SD. * p<0.05 **p<0.01 indicated significant difference vs. corresponding group(as shown in“Materials and methods”).(pIFN-γ_1_: a modified porcine gamma by yeast codon preference; pIFN-γ_1_-His tag: a modified porcine gamma by yeast codon preference (pIFN-γ_1_)with adding histidine label; pIFN-γ_1_’: pIFN-γ_1_ with C-terminal amino acid mutation; pIFN-γ_1_’-His tag: pIFN-γ_1_ with C-terminal amino acid mutation and adding histidine label).

The strains were inoculated on BMGY containing 0.5% methanol, and the bacteria were collected at 30°C, 25°C, 20°C and 15°C. Cells were lysed, the expression of pIFN-γ protein was detected by SDS-PAGE, and the content of pIFN-γ protein was detected by BCA protein assay (Fig 4A). The effect of different temperature fermentations on the expression of pIFN-γ in *Pichia pastoris* showed that the expression of pIFN-γ protein at 25°C was significantly higher than at 15°C and 20°C (*p*<0.05), especially for pIFN-γ_1_’ and pIFN-γ_1_’-His tag. After 25°C fermentation, the pIFN-γ protein content of the recombinant yeast was up to 773.4 mg/L and 848.2 mg/L, which was significantly higher than the protein produced by other fermentation temperatures (*p*<0.01). Therefore, the recombinant *Pichia pastoris* containing the optimized pIFN-γ gene with the 3’ end signal peptide mutation obtained a higher yield of pIFN-γ protein (cultivation for 0–24 h at 28°C and then 24 h-72 h at 25°C).

#### Fermentation with different DO concentrations in *Pichia pastoris*

The effect of different DO concentrations on the expression of pIFN-γ in *Pichia pastoris* was increased with the increase in DO concentration in a certain range, but when the concentration of DO exceeded 40%, the expression was significantly reduced (*p*<0.01). The expression of pIFN-γ protein was maximal when the concentration of DO in the substrate was 30%, especially for the recombinant *Pichia pastoris* with the optimized pIFN-γ gene and the 3’ signaling peptide mutation, which had significantly higher expression than that of the other treatment groups (*p*<0.05).

After 25°C fermentation, the content of protein of pIFN-γ1’ and pIFN-γ1’-His tag were up to 773.4 mg/L and 848.2 mg/L in shaker fermentation tank, when the concentration of DO in the substrate was 30%, the content of pIFN-γ1’ and pIFN-γ1’-His tag protein reached 8912 mg/L and 9557 mg/L in fermentation tank. From the above results, it can be seen that with fermentation tank fermentation, compared with shaker fermentation, yeast secretion of the porcine interferon increased significantly, while for the shaker fermentation, secretion increased 10 times and yeast wet weight also increased approximately 30%. The best fermentation conditions were 28°C, pH 6.0, and DO 50% from 0 to 24 h followed by 25°C, pH 6.0, and DO 30%, 24 h-72 h.

### Analysis of the biological activity of pIFN-γ

Purified pIFN-γ_1_, pIFN-γ_1_-His, pIFN-γ_1_’, and pIFN-γ_1_’-His protein were added to the IPEC-J2 cell culture solution at a final concentration of 10 μg/3 ml. The cells were cultured for 24 h at 5% CO2 and 37°C. Then, the cells were collected, and total RNA was extracted from the cells. *OAS1*, *Mx1* and *PKR* gene expression was detected in IPEC-J2 by qPCR after reverse transcription of the RNA.

#### The difference in *OAS1* expression levels after stimulation of different protein samples in IPEC-J2 cells

**Fig 5:**
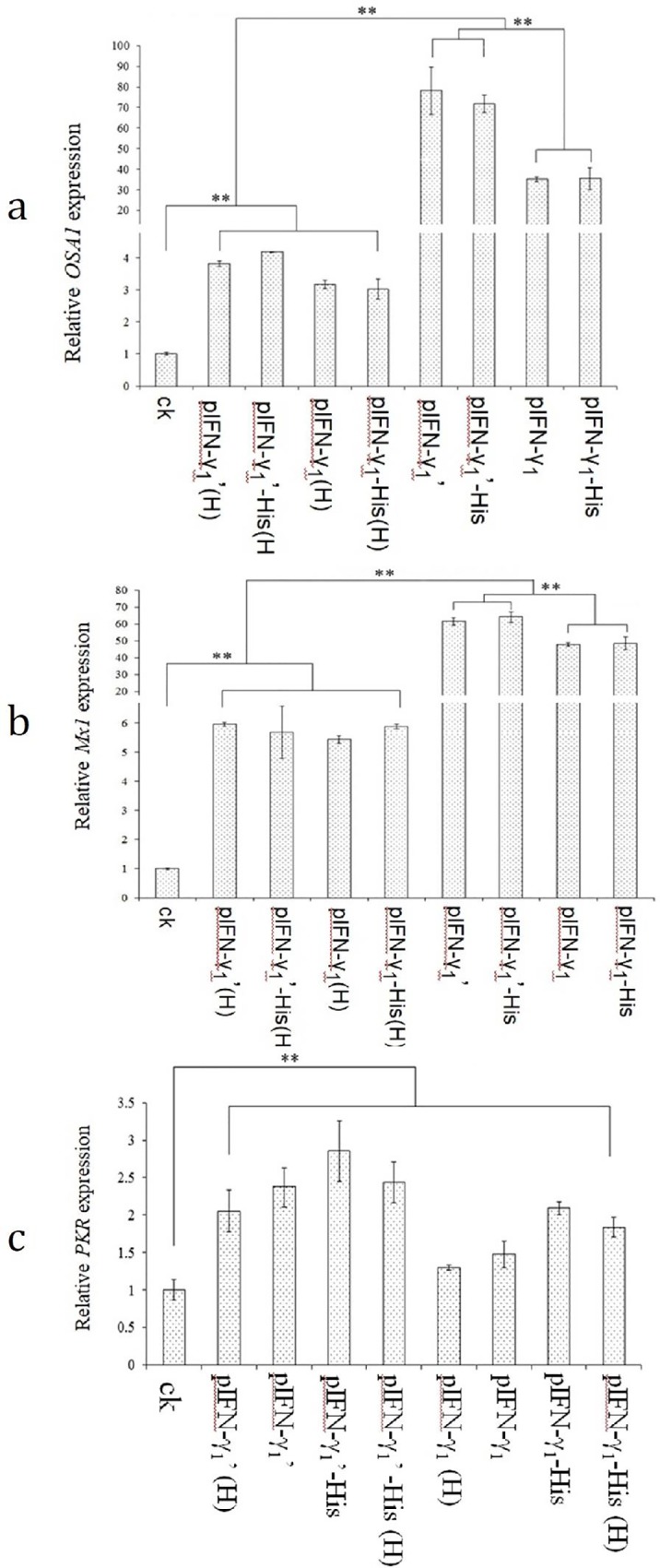
Analysis of biological activity of pIFN-r. (a) The difference of *OAS1* expression level after stimulation of different protein samples in cells were detected by qPCR. The expression level of *OAS1* in blank control cells was set as a reference. The expression level of *OAS1* in the cells of other different experimental treatment groups was a relative multiple of the blank treatment group. (b) The difference of *Mx1* expression level after stimulation of different protein samples in cells were detected by qPCR. The expression level of *Mx1* in blank control cells was set as a reference. (c) The difference of *PKR* expression level after stimulation of different protein samples in cells were detected by qPCR. The expression level of *PKR* in blank control cells was set as a reference. The results of the independent experiments were indicated as mean ±SD. * p<0.05 **p<0.01 indicated significant difference vs. control group (as shown in “Materials and methods”). (pIFN-γ_1_: a modified porcine gamma by yeast codon preference; pIFN-γ_1_(H): pIFN-γ_1_ with 80°C heating treatment for 30 min; pIFN-γ_1_-His tag: a modified porcine gamma by yeast codon preference (pIFN-γ_1_) with adding histidine label; pIFN-γ_1_(H)-His tag: pIFN-γ_1_-His tag with 80°C heating treatment for 30 min pIFN-γ_1_’: pIFN-γ1 with C-terminal amino acid mutation; pIFN-γ_1_’(H): pIFN-γ_1_’ with 80°C heating treatment for 30 min; pIFN-γ_1_’-His tag: pIFN-γ_1_ with C-terminal amino acid mutation and adding histidine label; pIFN-γ_1_’-His tag(H): pIFN-γ_1_’-His tag with 80°C heating treatment for 30 min).

The expression level of *OAS1* in the cells of each treatment group was significantly improved (*p*<0.05) (Fig 5A), especially in the pIFN-γ_1_’ and pIFN–γ_1_’-His tag treatment groups, which had 78.17 times and 71.74 times increased expression levels of *OAS1*, respectively (*p*<0.01). Meanwhile, pIFN-γ_1_’, pIFN-γ_1_’-His tag, pIFN-γ_1_ and pIFN-γ_1_-His tag treatment groups had significantly higher expression of *OAS1* in cells than the control group (p<0.01). It was also found that the expression level of *OAS1* in the treatment groups, pIFN-γ_1_’ and pIFN-γ_1_’-His tag, was significantly higher than that of pIFN-γ_1_ and pIFN-γ_1_-His tag in the processing group by as much as two times (p<0.05), but the difference in the corresponding group was not obvious. The pIFN-γ expressed in each optimized treatment group increased the expression of *OAS1* after 80°C treatment for 30 min, which was significantly higher than that of the control group (*p*<0.05) but was significantly lower than that of the group without heat treatment.

#### The difference in *Mx1* expression levels after stimulation of different protein samples in IPEC-J2 cells

The expression level of *Mx1* in the cells of each treatment group was significantly improved (*p*<0.05) (Fig 5B), especially in the pIFN-γ_1_’ and pIFN-γ_1_’-His tag treatment groups, which had 61.39 times and 64.13 times increased expression levels of *Mx1*, respectively (p<0.01). Meanwhile, the pIFN-γ_1_’, pIFN-γ_1_’-His tag, pIFN-γ_1_ and pIFN-γ_1_-His tag treatment groups had significantly higher *Mx1* expression than the group with 80°C heating for 30 min (*p*<0.01). It was also found that the expression level of *Mx1* in pIFN-γ_1_’ and pIFN-γ_1_’-His of the tag treatment group was significantly higher than that of pIFN-γ_1_ and pIFN-γ_1_-His in the tag processing group (p<0.05), but the difference in the corresponding group was not obvious.

#### Differences in *PKR* expression levels after stimulation of different protein samples in IPEC-J2 cells

As shown in Fig 5C, all recombinant expression of the pIFN-γ protein was increased compared to the blank control, and the expression levels of *PKR* in the eight treatment groups were increased significantly compared to the blank control group (p<0.05), but there was no significant difference between each experimental group and its corresponding heat- treatment group.

## Discussion

TianYuan et al., studies have found that when the pig interferon gene was transferred to the prokaryotic carrier, the expressed protein station is 40%, mainly in the form of inclusion body, and the purification activity is affected to a certain extent[24]. The *Pichia pastoris* expression system is an excellent eukaryotic expression system developed in recent years: it has a strong AOX promoter, an exogenous protein that has a certain function in translation modification, and the advantages of primitive nuclear biological molecular genetics and simple operation. To date, more than 1000 exogenous proteins[25] have been successfully expressed by the *Pichia pastoris* expression system. However, there are some defects in the expression system of *Pichia pastoris*: some exogenous proteins are easily degraded by *Pichia pastoris*, there are still some exogenous proteins in the system that cannot be expressed or have low expression, and some exogenous proteins may have excessive glycosylation modification.

A large number of studies have shown that the appropriate and targeted optimization of exogenous genes is of great significance for improving their expression level[26–27]. Sreekrishna et al.[28] found that the expression level of human serum albumin mRNA was increased more than 50 times after it was optimized, similar to that of AOX1 mRNA. Xu et al.[29] found that the xylanase gene optimized by a yeast-preferred codon could significantly improve its expression level. Hu et al.[30] studied the expression of T-cell immune toxin by *Pichia pastoris* and found that the cDNA sequence was rich in A/T bases, which led to the early termination of transcription. After the A/T content and distribution were adjusted by codon optimization, the target protein was successfully expressed. In this study, the gene sequence optimization software developed by the State Key Laboratory of Pest Control and Resource Utilization at Sun Yat-Sen University, the COStar optimization tool, was combined with the optimization software Eugene, the mRNA local energy and the *Pichia pastoris* codon optimization table to comprehensively reconstruct the pIFN-γ coding sequence. There was a certain amount of exogenous protease expression inside and outside of the cells of the host bacterium *Pichia pastoris*, so that most exogenous proteins were subject to degradation during both intracellular expression and secretory expression, which was also an important factor affecting the expression. Degradation led to a decrease in the yield of the target protein, and the fragments formed by degradation also caused great difficulty in separation and purification, especially for the degradation products with small differences in the molecular weight of the target protein. Because of their physical and chemical properties, the conventional separation method was very difficult to use for their separation, resulting in greatly reduced product yield, low product purity, and decreased activity of the protein[31]. The intracellular protease mainly involves the degradation of protein precursors to produce active proteins, and the removal of protein signaling peptides after the transfer to the membrane leads to protein inactivation. By analyzing the C-terminal sequence of the pIFN-γ protein, it was found that the LRKRKRS sequence of the C-terminal amino acid is similar to the signal peptide sequence (EKREAEAE) and is easily enzymolysed by the signal peptide enzyme, resulting in degradation and inactivation of the pIFN-γ protein. Therefore, we mutated the arginine at positions 129 and 131 of the C-terminal end of the pIFN-γ protein to histidine (LRKRKRS mutation to RKHKH), thereby allowing the pIFN-γ protein to be secreted without degradation, and we added a histidine tag at the end of the C-terminus for easy purification and further research.

The temperature had an effect on the activity of yeast fermentation product, the physical properties of fermentation broth and the direction of biosynthesis[32]. The culture temperature of *Pichia pastoris* is suitable between 28~30°C, but 28°C is the optimum temperature for yeast body growth. It was found that the proper temperature reduction of *Pichia pastoris* is beneficial to the secretion of exogenous proteins, which may be due to the high temperature formation of disulfide bonds, which altered the structure of target proteins and seriously affected protein activity and yield[23]. At the same time, dissolved oxygen in the fermentation substrate was very important for the growth of aerobic microorganisms. In the process of fermentation, especially for *Pichia pastoris*, ensuring that the supply of oxygen met the demand for oxygen was an important factor for improving the fermentative yield. In earlier studies, the expression level of pIFN-γ in *Pichia pastoris* was 108 mg/L. Compared with that, the expression level of pIFN-γ in this study was up to 9557 mg/L[33–34]. By using a fermentation tank for large-scale fermentation, the expression level of exogenous protein could be increased 10~100 times that of a common shaker because the ventilation of flask fermentation was not ideal, and the density of fungal growth and the expression of exogenous protein were strongly affected. It was found that in the process of large-scale fermentation of *Pichia pastoris*, if the DO is less than 20%, it will affect growth and metabolism. If the DO is more than 60%, the cells will have oxygen poisoning. Therefore, the effective control of DO in large-scale fermentation is very important for the expression of exogenous proteins[21]. Through our study on the fermentation conditions of gradient temperature and gradient DO, it was found that the recombinant *Pichia pastoris* strain could obtain a high yield of pIFN-γ protein after stepwise variable temperature fermentation (0–12 h incubation at 28°C and 50% DO and 24 h-72 h of incubation at 25°C, pH 6.0 and 30% dissolved oxygen, up to 9536 mg/L). Meanwhile, analysis of porcine interferon activity by qPCR showed that the expression of the *OAS1* and *Mx1* genes in IPEC-J2 cells could be significantly improved by the pIFN-γ gene C-terminal mutation in 4 strains. The target proteins upregulated by the C-terminal peptide-specific amino acid mutations are more likely to be stimulated by the cells. We speculated that the integrity of the C-terminus of the pIFN-γ peptide chain is important for the formation or maintenance of the pIFN-γ spatial two-polymer structure. The structure of the C-terminal end was not complete, which resulted in the decreased activity of pIFN-γ. After the C-terminal mutation of the pIFN-γ sequence, the secreted expression of pIFN-γ protein contained a complete peptide chain structure and maintained high biological activity. Meanwhile, we also found that the pIFN-γ protein expressed by gene optimization was still active after high temperature treatment, indicating that the C-terminal mutation strategy is beneficial to the maintenance of the stability and biological activity of the pIFN-γ protein structure.

In conclusion, in this study, *Pichia pastoris* was used as the expression system, the optimized pIFN-γ gene sequence was used as an inserting fragment, and high copy secretory expression strains of pIFN-γ_1_, pIFN-γ_1_-His tag and pIFN-γ_1_’ and pIFN-γ_1_’-His tag *Pichia pastoris* were constructed successfully. The functional differences between 4 different proteins were compared at the cell level to analyze their stimulating activity, and the processing mechanism and optimal expression strategies of exogenous proteins secreted in *Pichia pastoris* were studied. The results showed that the C-terminal signaling peptide gene mutation and the addition of a histidine tag in pIFN-γ resulted in a highly expressed pIFN-γ protein that could be obtained in *Pichia pastoris* with higher bioactivity. It was also shown that when recombinant *Pichia pastoris* pIFN-γ was cultured for 0–24 h at 28°C and 50% DO, and then for 24–72 h at 25°C and 30% DO, higher secretion (up to 10 times compared to shake flask fermentation) was achieved while maintaining good living conditions.

## Ethical approval

This article does not contain any studies with human participants or animals performed by any of the authors.

## Supporting information

S1 Fig Substitution omega PCR for pPICZαA –γ_1_ construction. (DOC)

S2 Fig Insertion omega PCR for pPICZαA- pIFN-γ_1_-His construction. (DOC)

S3 Fig Mutation of the enzyme-cut point of pIFN-γ gene 3’ C-terminal α-signaling peptide. (DOC)

S4 Fig The plasmids used in this study. (DOC)

S1 Table The plasmids used in this study. (DOC)

S2 Table Primers for PCR reactions. (DOC)
