# Supplementary material for: Modulating the 3’ end-DNA and the fermentation process for enhanced production and biological activity of porcine interferon-gamma
Source: PLoS One. 2019 Mar 26;14(3):e0214319. doi: 10.1371/journal.pone.0214319 (PMC6435167; doi:10.1371/journal.pone.0214319)
Supplement: S1 Fig — (DOC) [file pone.0214319.s001.doc]

**Final**

**plasmid**

***Xho1***

***Xba*1**

pPICZαA

pPICZαA

**Starting**

**plasmid**

**Chimeric**

**primers**

**First PCR**

**products**

**plasmid**

**Omega-PCR**

**Substitution mode**

**plasmid**

**pIFN-γ_1_ (Zeo)**

***Xho*1**

***Xba*1**

**pIFN-γ_1_ (Zeo)**

**pIFN-γ_1_ (Zeo)**

pPICZαA

**Zeo**

F-pIFN-γ-*Xho*Ⅰ

R-pIFN-γ-*Xba*Ⅰ

**S1 Fig. Substitution omega PCR for pPICZαA –γ_1_ construction**
