# Supplementary material for: Modulating the 3’ end-DNA and the fermentation process for enhanced production and biological activity of porcine interferon-gamma
Source: PLoS One. 2019 Mar 26;14(3):e0214319. doi: 10.1371/journal.pone.0214319 (PMC6435167; doi:10.1371/journal.pone.0214319)
Supplement: S2 Fig — (DOC) [file pone.0214319.s002.doc]

**Final**

**plasmid**

R-pIFN-γ-*Xba*Ⅰ

F-pIFN-γ-*Xho*Ⅰ

R-pIFN-γ-His-*Xba*Ⅰ

pIFN-γ_1_

pIFN-γ_1_-His

**Starting**

**plasmid**

**pIFN-γ_1_-His** (Zenocin)

**Omega-PCR**

**Insertion mode**

**plasmid**

**Final**

**plasmid**

*Xho*1

*Xba*1

***Xho*1**

***Xba*1**

pPICZαA

pPICZαA

**Chimeric**

**primers**

**First PCR**

**products**

**plasmid**

pPICZαA

**S2 Fig. Insertion omega PCR for pPICZαA- pIFN-γ_1_-His construction**
