# Supplementary figures and images for: Modulating the 3’ end-DNA and the fermentation process for enhanced production and biological activity of porcine interferon-gamma
Source: PLoS One. 2019 Mar 26;14(3):e0214319. doi: 10.1371/journal.pone.0214319 (PMC6435167; doi:10.1371/journal.pone.0214319)

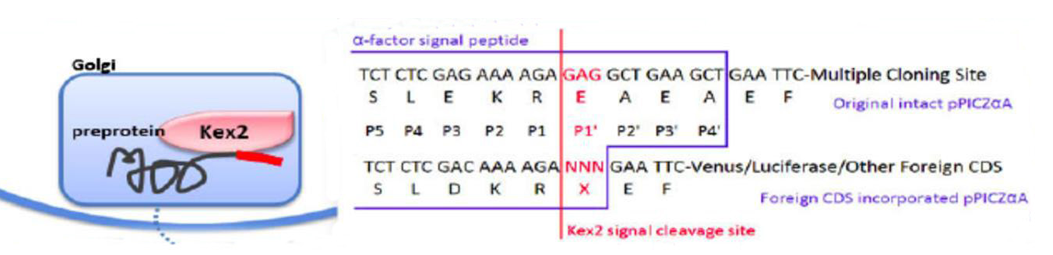


**S3 Fig. Mutation of the enzyme-cut point of pIFN-γ gene 3' C-terminal α-signaling peptide**

Supplement: S3 Fig — (DOC) [file pone.0214319.s003.doc]

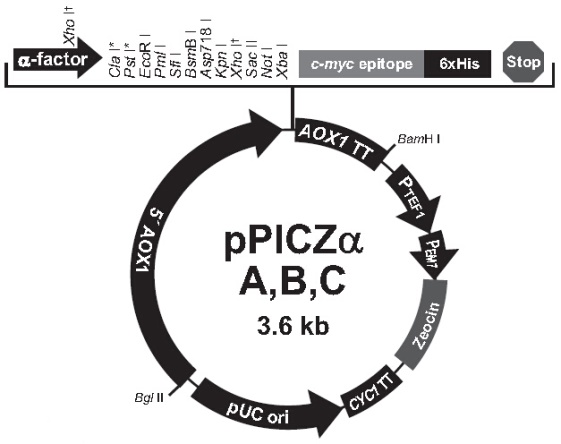


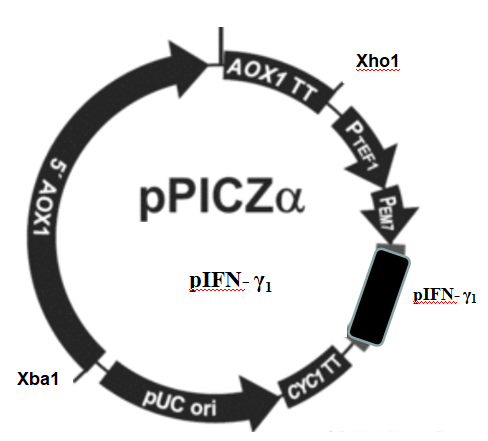

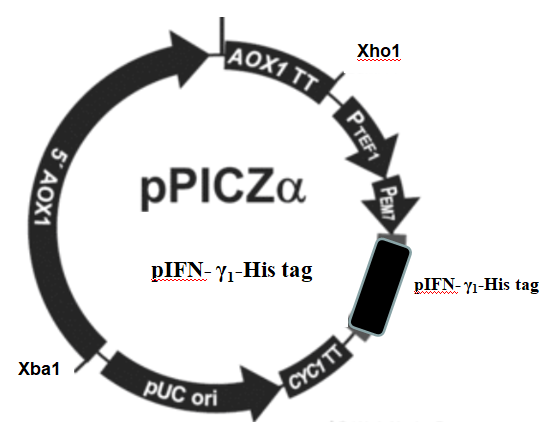


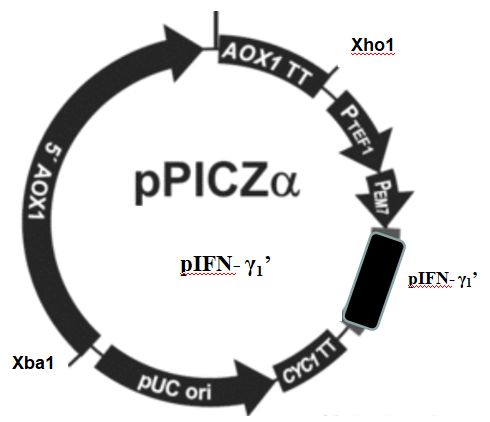

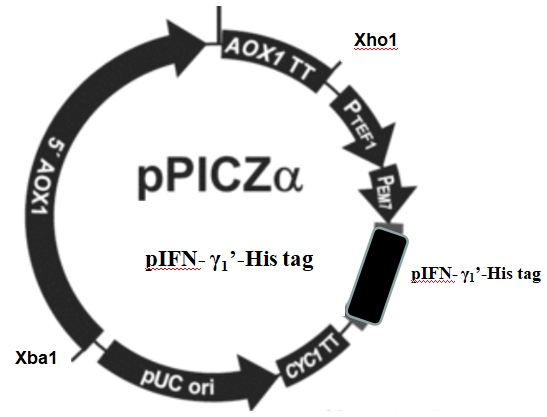


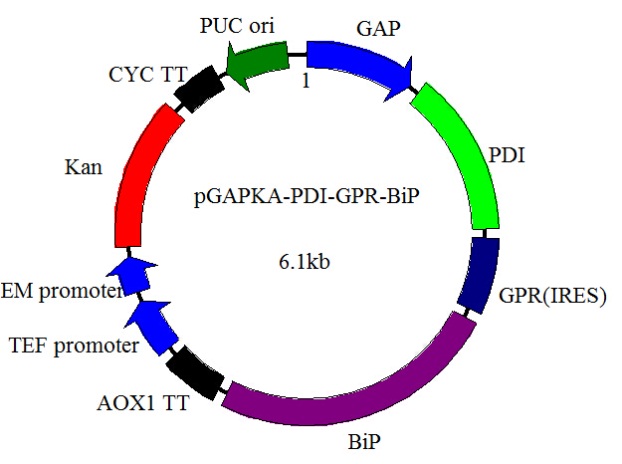


Fig. S3 The plasmids used in this study

**S4 Fig. The plasmids used in this study**

Supplement: S4 Fig — (DOC) [file pone.0214319.s004.doc]
