# Supplementary material for: Modulating the 3’ end-DNA and the fermentation process for enhanced production and biological activity of porcine interferon-gamma
Source: PLoS One. 2019 Mar 26;14(3):e0214319. doi: 10.1371/journal.pone.0214319 (PMC6435167; doi:10.1371/journal.pone.0214319)
Supplement: S1 Table — (DOC) [file pone.0214319.s005.doc]

**S1 Table. The plasmids used in this study**

| **Plasmids Marker** | |
| --- | --- |
| pPICZɑA | Zeocin |
| pPICZɑA- pIFN-γ_1_ | Zeocin |
| pPICZɑA- pIFN-γ_1_-His tag | Zeocin |
| pPICZαA-pIFN-γ_1_ ́ | Zeocin |
| pPICZαA-pIFN-γ_1_ ́-His tag | Zeocin |
