# Supplementary material for: Modulating the 3’ end-DNA and the fermentation process for enhanced production and biological activity of porcine interferon-gamma
Source: PLoS One. 2019 Mar 26;14(3):e0214319. doi: 10.1371/journal.pone.0214319 (PMC6435167; doi:10.1371/journal.pone.0214319)
Supplement: S2 Table — (DOC) [file pone.0214319.s006.doc]

**S2 Table. Primers for PCR reactions**

| Name | Sequences (5'−3') |
| --- | --- |
| 5’AOX | GACTGGTTCCAATTGTTGACAAGC |
| 3’AOX | GCAAATGGCATTCTGACATCC |
| P1 | CAAGCTCCATTCTTCAAGGAGATCACTATCCTGAAGGACTACTTCAACGCTTCAACTTC |
| P2 | AGGATCTCCAGGAATAATGGACCACCGTTAGGAACATCTGAAGTTGAAGCGTTGAAGTA |
| P3 | CCATTATTCCTGGAGATCCTGAAGAATTGGAAGGAAGAAAGTGACAAGAAGATCATCCA |
| P4 | ATCTCGAAGAACTTGAAGTAGAATGAGACGATTTGTGATTGGATGATCTTCTTGTCACT |
| P5 | ACTTCAAGTTCTTCGAGATCTTCAAGGACAACCAAGCTATCCAGAGATCAATGGACGTT |
| P6 | AGAACCGTTCAAGAATCTTTGGAACATGTCCTGCTTGATAACGTCCATTGATCTCTGGA |
| P7 | AAAGATTCTTGAACGGTTCTTCCGGAAAGTTAAACGATTTCGAGAAGCTGATCAAGATC |
| P8 | GAGATAGCCTTTCTCTGGATTTGCAAGTTGTCGACGGGGATCTTGATCAGCTTCTCGAA |
| P9 | ATCCAGAGAAAGGCTATCTCAGAGTTGATCAAGGTCATGAACGATTTGAGTCCAAGATC |
| P10 | GGTTTGACTACGCTTTCTCTTTCTCAGGTTTGATCTTGGACTCAAATCGTTC |
| P11 | AGAGAAAGCGTAGTCAAACCATGTTCCAAGGTCAAAGAGCTTCAAAGTAG |
| P12 | CTACTTTGAAGCTCTTTGA |
| P10´ | GGTTTGACTATGCTTATGCTTTCTCAGGTTTGATCTTGGACTCAAATCGTTC |
| P11´ | AGCATAAGCATAGTCAAACCATGTTCCAAGGTCAAAGAGCTTCAAAGTAG |
| F-pINF-γ | CAAGCTCCATTCTTCAAGGAGATCA |
| R-pIFN-γ | CTACTTTGAAGCTCTTTGACC |
| F-pINF-γ-*Xho*Ⅰ | CCGCTCGAGAAGAGAGAGGCTGAAGCTCAAGCTCCATTCTTCAAGGAGATCA |
| R-pIFN-γ-*Xba*Ⅰ | GCTCTAGACTACTTTGAAGCTCTTTGACC |
| R-pIFN-γ-H-*Xba*Ⅰ | GCTCTAGACTAATGATGATGATGATGATGCTTTGAAGCTCTTTGACC |
| F-*β-actin* | TCTGGCACCACACCTTCT |
| R-*β-actin* | TGATCTGGGTCATCTTCTCAC |
| F-*PKR* | GAGAAGGTAGAGCGTGAAG |
| R-*PKR* | CCAGCAACCGTAGTAGAG |
| F-*Mx1* | TAGGCAATCAGCCATACG |
| R-*Mx1* | GTTGATGGTCTCCTGCTTAC |
| F-*OAS1* | AAGTGACGTTTGAGGTCCAGAG |
| F-*OAS1* | GGACGTAGATTTCCGGGTTGG |
